# P22 Phage Shows Promising Antibacterial Activity under Pathophysiological Conditions

**DOI:** 10.26502/ami.93650078

**Published:** 2022-02-10

**Authors:** Logan Gildea, Joseph A. Ayariga, Boakai K. Robertson, Robert Villafane

**Affiliations:** 1 The Microbiology Program, College of Science, Technology, Engineering and Mathematics (C-STEM), Alabama State University, Montgomery, AL, 36104; 2 The Biomedical Engineering Program, College of Science, Technology, Engineering and Mathematics (C-STEM), Alabama State University, 1627 Hall Street, Montgomery, AL, 36104; 3 Department of Biological Sciences, Microbiology PhD. Program, College of Science, Technology, Engineering and Mathematics (C-STEM), Alabama State University, 1627 Hall Street Montgomery, Alabama 36104

**Keywords:** Bacteriophage therapy, antimicrobial resistance, *Salmonella*, antibiotic synergy

## Abstract

The prevalence of multidrug resistant bacterial diseases is a major global health risk. Multidrug resistant bacterial diseases are prevalent, and the need for novel methods of treatment is essential to the preservation of public health. Annually foodborne pathogens cause 1.35 million infections and 26,500 hospitalizations in the United States alone. Foodborne pathogens such as *Salmonella* spp. are a major threat to public health. Bacteriophages offer a unique method for the treatment of these multidrug resistant bacteria. We studied the infection dynamics of a potential mono-phage therapy of *Salmonella typhimurium* under various pathophysiological conditions. Furthermore, we determined the resistance dynamics of *Salmonella typhimurium* against P22 phage treatment. We also determined synergy with antibiotics such as ampicillin and kanamycin. This research helps to further define and show the versatility of bacteriophages as potential novel treatment methods.

## 1. Introduction

The discovery of antibiotics revolutionized modern medicine; however, as we progress into the mid-21^st^ century, the prevalence of antibiotic-resistant bacteria is a major threat to public health. The massive and sometimes inappropriate use of antibiotics has led to the increased prevalence of multidrug-resistant bacterial strains that present treatment challenges in the clinical setting [1–4]. Antibiotic-resistant bacterial infections account for roughly 2.8 million infections and 35,000 deaths annually in the United States alone [2]. These infections pose serious threats to public health and are also a major economic burden costing an estimated 7.7 billion dollars in medical costs annually [5]. A major hazard to public health is the increasing prevalence of antibiotic and multidrug-resistant foodborne pathogens. As these infections are becoming increasingly prevalent, the development of new antibiotic agents has declined, creating a major problem in the field of medicine [6, 7].

One of the most prevalent foodborne pathogens is *Salmonella*, which can be found in numerous food products such as poultry, seafood, and other fresh and processed meats [8–11]. Annually *Salmonella spp*. accounts for 1.35 million infections, 26,500 hospitalizations, and 420 deaths in the United States alone. An increased prevalence of antibiotic-resistant *Salmonella* strains could potentially result in an increase rate of mortality making it critical to develop new novel treatment strategies. *Salmonella typhimurium* has been of significant relevance due to the reported prevalence of antibiotic-resistance in this species [12, 13]. The Centers for Disease Control (CDC) reported in 2019 that *Salmonella typhimurium* accounted for 59% of ampicillin-resistant infections [2]. Continued mass usage of general antibiotics could drastically increase the prevalence of antibiotic resistant *S. typhimurium* as well as other *Salmonella* species [5, 14].

A potential alternative to antibiotic treatment is the utilization of phage therapy as a novel therapeutic treatment [15–18]. Bacteriophages, commonly referred to as phages, are prokaryotic viruses that infect bacterial species with high specificity. An important distinction of phages is their lifestyle which can either be lytic or lysogenic. The use of phages featuring the lytic phenotype is advised for the development of therapeutics [19, 20]. The host specificity of phages is a major benefit of phage therapy in comparison to traditional treatment methods such as antibiotics. The high host specificity of phages allows them to only interact with the target pathogen while leaving natural microbiota and eukaryotic cells unaffected [21, 11]. An additional benefit of phage therapy is the suggested synergy between phages and antibiotics that allows for co-therapy [15–18]. Co-therapy refers to the use of both antibiotics and phages in synonymy for the treatment of resistant or reoccurring bacterial infections. This method relies on the inability of the bacterial species to rapidly develop resistance to both treatment methods and utilizes the synergy that is sometimes present between antibiotics and phages [22–24]. Co-therapy has been suggested in literature as a potentially viable method for the treatment of multidrug-resistant bacterial strains [15–18].

Phage therapy is a strong potential candidate for the treatment of foodborne pathogens such as *Salmonella typhimurium*. The lytic *Salmonella* phage P22 has been widely studied but requires further study to be utilized as a standard treatment against *Salmonella typhimurium* [25, 26, 11]. Our study provides insight into the pathophysiological characteristics that the phage P22 must withstand to be considered a viable therapeutic against *Salmonella typhimurium*. *Salmonella typhimurium* typically colonizes within the small or large intestines after the pathogen is consumed [28]. The variation of pH throughout these organs is a challenge in the development of antibacterial agents [29, 30]. Determination of P22’s efficacy in pathophysiological conditions similar to the small and large intestines is critical to its progression as a potential therapeutic. Designing a phage therapy method for *Salmonella typhimurium* would also require further understanding of phage-host interactions in disease-mimicking conditions.

In this study, we have performed phage-bacteria kinetic studies under several pathophysiological conditions related to *Salmonella typhimurium* infections. These pathophysiological conditions include acidic pH and stationary phase. We utilized *Salmonella typhimurium* (referred to as BV4012) as the model organism for this study. This strain has a generation time of 30 minutes, allowing for kinetics under pathophysiological conditions to be examined rapidly. We show the ability of the phage P22 as an effective antibacterial agent against *Salmonella typhimurium* under low pH and stationary conditions. Additionally, we showed synergy with ampicillin and kanamycin. Most importantly, we showed that P22 could prevent the growth of *Salmonella typhimurium* under pathophysiological conditions, encouraging further study of P22 and other similar phages as potential antibacterial agents.

## 2. Methods

### 2.1 Soft Agar Overlay and Spot Assays for Phage Enumeration

To detect and quantify the phage P22, the soft agar overlay technique was utilized. Soft Agar was prepared by adding agar (0.8%) to Luria-Bertani (LB) media. (BD Difco, USA) This suspension was autoclaved and allowed to cool to 45 °C in a water bath. 1 mL of late log phase BV4012 bacterial culture (OD > 1) and 100 μL of P22 phage dilutions were added to 5 mL of the soft agar and was poured onto a Luria agar plate. The soft agar was allowed to cool to room temperature and solidify. These plates were then incubated at 37 °C overnight.

For verification of phage titers, spot assays were conducted using late log phase *Salmonella typhimurium* BV4012 (OD > 1). These cultures were plated on a LB agar plate to form a bacterial lawn. P22 phage samples were serially diluted and spotted onto the bacterial lawn. Plates were incubated at 37 °C for 24 h, and the number of plaques formed at various dilutions was quantified

### 2.2 Phage Amplification and Maintenance

P22 phage was amplified on *S. typhimurium* host. Liquid amplification was carried out by the addition of a P22 sample at 10 MOI to a log phase culture (OD between 1 and 2) and incubated for 12–24 h at 37 °C with rotary shaking at 180 rpm. Following incubation cultures were then centrifuged at 4,000 g for 10 minutes, and the supernatant was collected. For plate amplification, a soft agar overlay containing log phage *S. typhimurium* (OD 1–2) was prepared, and the P22 phage sample (10 MOI) was spotted on top of the soft agar. The plates were incubated for 12–24 h at 37 °C. The soft agar overlay was used to determine phage titers [31].

### 2.3 Bacterial Growth Kinetics

To study bacterial growth kinetics, LB media was prepared and inoculated with 50 μL of lag-phase secondary bacterial culture (OD<1) per 10 mL of the media. The cultures were then incubated at 37 °C with rotary shaking at 180 rpm. Measurements of bacterial density (OD) were taken periodically over the span of 48 h [31].

### 2.4 Bacterial Growth Kinetics in the Prescence of P22 Phage- pH and Stationary Phase

To study how pathophysiological conditions affect infection dynamics, phage kinetics assays were performed to observe phage kinetics at acidic pH and stationary phase. To study the effects of pH on phage infection dynamics, a phage kinetics assay was performed at varied media pH. The pH of the media was reduced from 6.8 utilizing Hydrochloric acid to either 6.0 or 5.5. Bacteria was inoculated into the media at pH 6.8, 6.0, and 5.5. Phage treated samples were inoculated with P22 phage at a MOI of 10 at the beginning of the experiment (lag phase).

To examine phage kinetics in the stationary phase, BV4012 (Salmonella typhimurium) was grown in LB media until the OD was 10. The stationary phase cultures were then either treated with the phage P22 at 10 MOI or left untreated. OD values were obtained periodically over the span of 48 h.

### 2.5 Antibiotic Minimum Inhibitory Concentration (MIC) Determination

The MICs of ampicillin and kanamycin were determined to establish the antibacterial activity of the respective antibiotics against Salmonella typhimurium strains (BV4012). Antibiotic stock solutions of 50 ug/mL in sterile ddH_2_O were made for these sets of experiments. Stocks were stored in a −20 °C refrigerator until usage. Several dilutions were prepared in serial fashion (50 μg/mL, 5 μg/mL, 0.5 μg/mL, 0.05 μg/mL). A 96 well plate with 100 μL of Salmonella typhimurium culture with an OD of 0.1 in each well was inoculated with either one of the antibiotic dilutions or left untreated. The plate was incubated for 48 h at 37 °C and growth was measured through absorbance at 600 nm. The lowest concentration of antibiotic without bacterial growth was termed as the minimum inhibitory concentration (MIC) [31].

### 2.6 Antibiotic Growth Kinetics

To study the effects of the antibiotic ampicillin or kanamycin on Salmonella typhimurium growth. LB media was prepared and inoculated with 50 μL of log phase secondary bacterial culture per 10 mL of the media. The cultures were then incubated at 37 °C with rotary shaking at 180 rpm. The OD of the bacteria was periodically measured against a media blank. The final concentrations of antibiotics in treated samples were either 50 μg/mL or 5 μg/mL were added at the beginning of the experiment (lag phase). To study the possible synergetic relationship between P22 phage and the antibiotics used, cultures were co-treated with an antibiotic and the P22 phage at MOIs of 10, 1, and 0.1 [31].

### 2.7 Phage Rechallenge Experiments

To determine the susceptibility of bacterial populations and developed resistance after exposure to the phage P22, BV4012 cultures were seeded into a 96 well plate (Fisherbrand™, FisherScientific, USA) and inoculated initially with P22, ampicillin, or kanamycin. The OD600 was recorded over a period of 48 h. Bacterial cultures that showed developed resistance following 48 h of exposure to phage or antibiotic treatment were treated again with P22. The OD600 was recorded following rechallenge for 24 h.

### 2.8 Phage-Antibiotic Synergy Assay

To study the potential synergy between ampicillin or kanamycin and the phage P22, the checkerboard assay was utilized. Salmonella typhimurium strain (BV4012) was seeded into a 96 well plate at an OD of 0.01 (50 μL/10 mL of media). Plate cultures were then inoculated with antibiotic and phage at respective concentrations and incubated at 37 °C while shaking at 180 rpm for 24 h. OD readings were taken at 600 nm using a plate reader (Biotek Cytation 3 Cell Imaging System). Fractional Inhibitory Concentrations (FIC) values were calculated using the formula ∑FIC = FICAB + FICP22 = (CAB/MICAB) + (CP22/MICP22) [31, 32]. In this calculation, MIC_AB_ and MIC_p22_ are representative of the MIC values of the antibiotic used and the MIC value of P22. Values <0.5 were defined as synergistic, values between 0.5 and 4 as no interaction or additive, and values over four as antagonism.

### 2.9 Phage Stability Assay

To study the stability of the phage P22 at varying pH, a 10^8^ solution of P22 phage at pH 6.8, 6.0, and 5.5 were incubated at 37 °C. Samples of the solutions were collected and titrated against S. typhimurium.

### 2.10 SEM Observation

BV4012 samples were treated with P22 phage, and the morphology and surface microstructure of the bacteria observed using scanning electron microscopy. Untreated BV4012 served as control. Prior to microscopy, the specimens were dried in a vacuum, and sprayed with gold using EMS Quorum (EMS 150R ES) ion-sputtering instrument and observed through Analytical Scanning Electron Microscope (SEM) (JEOL JSM-6010LA, Japan) installed with IntouchScope software.

Sample preparation for SEM was carried out as described by Li et al., 2021 [41] with few modifications. In short, the specimens for SEM were fixed with 10% formaldehyde solution at room temperature for 10 min, washed with PBS solution thrice, and dehydrated serially in 50%, 70%, and 95% absolute ethanol solutions for 10 min each. Finally, the specimens were dried in a vacuum, and sprayed with gold using EMS Quorum (EMS 150R ES) ion-sputtering instrument and observed through Analytical Scanning Electron Microscope (SEM) (JEOL JSM-6010LA, Japan) installed with IntouchScope software.

### 2.11 Statistical Analysis

All experiments were performed on independent biological replicates. Statistical significance was determined for control and experimental groups using paired sample t-test. Data points were excluded if contamination was identified.

## 3. Results

### 3.1 P22 Phage Reduces Bacterial Growth of Salmonella typhimurium

**Figure 1:**
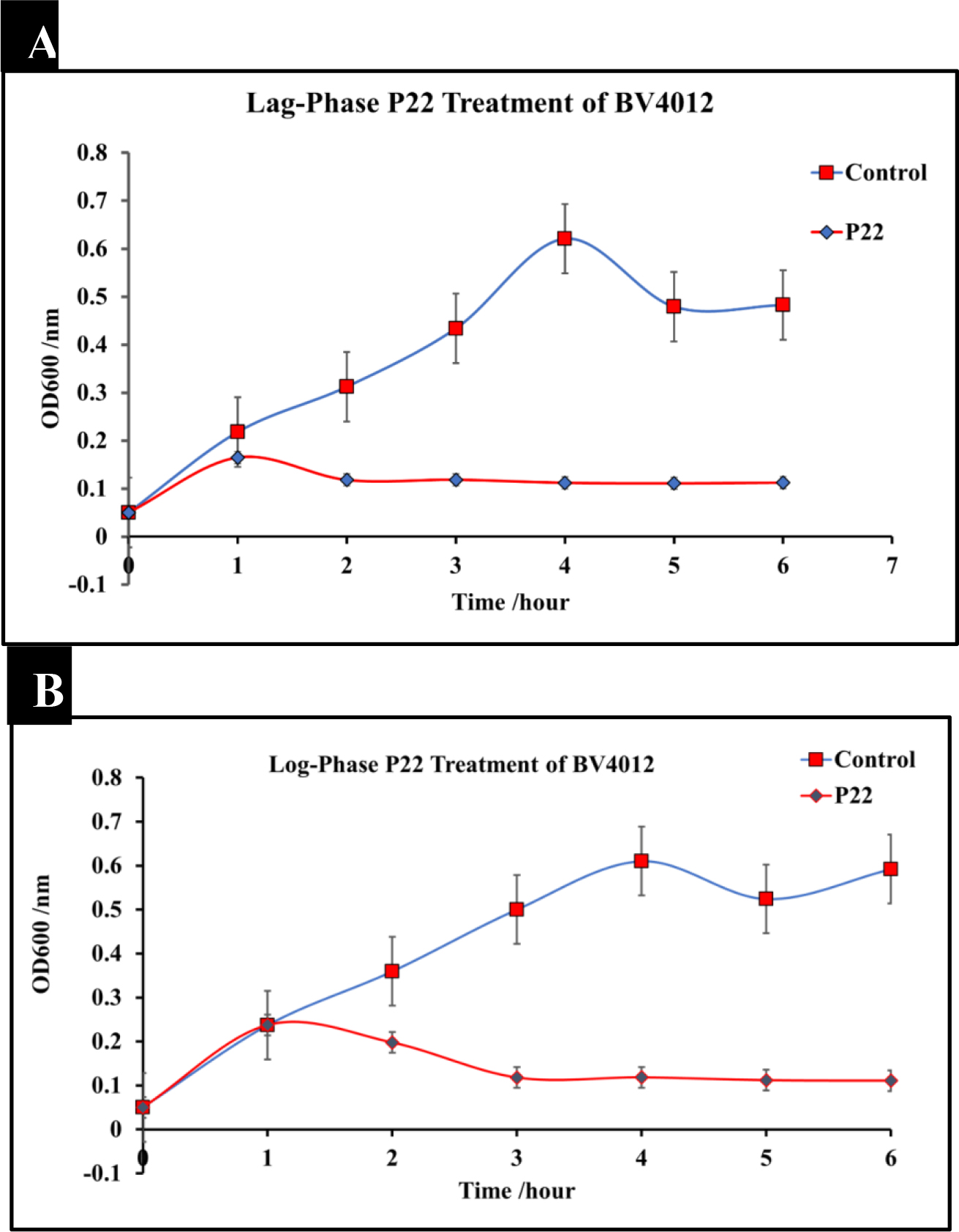
Treatment of *S. typhimurium* at lag and log phase with P22 phage. *S. typhimurium* cultures (BV4012) were treated with P22 phage at either the lag or log-phase. Three replicate OD600 were recorded hourly for six hours to examine the effect of P22 phage treatment on bacterial growth. **(A)** Lag-Phase P22 phage treatment of BV4012. **(B)** Log-Phase P22 phage treatment of BV4012.

The phage P22 was amplified to 10^9^ pfu/mL. To determine the effect of P22 phage on *Salmonella typhimurium* culture (BV4012), cultures were infected with P22 phage at the MOI of 10 either in the lag-phase or in the mid-log-phase. The optical density (OD) was recorded using a spectrometer (Molecular Devices SpectraMax® ABS Plus) at various time intervals. *S. typhimurium* cultures treated with P22 phage showed a significant reduction in OD in the mid-log phase as well as an inhibition of growth at the lag phase within the initial 8 h following infection (Fig. 1). Bacteria commonly develop resistance after extended exposure to agents such as antibiotics and other various antimicrobials. Our kinetics studies followed *S. typhimurium* strains development of resistance against P22 phage treatment. Bacterial regrowth was observed 48 h after treatment with P22 phage suggesting the development of resistance against P22 by both strains of *S. typhimurium*. Bacterial strains were rechallenged with an identical P22 phage treatment at the MOI of 10 following 48 h to observed susceptibility. An observed decrease in optical density indicated that there was no significant reduction in susceptibility to the P22 phage treatment.

### 3.2 SEM analysis of P22 Phage treatment of S. typhimurium

**Figure 2:**
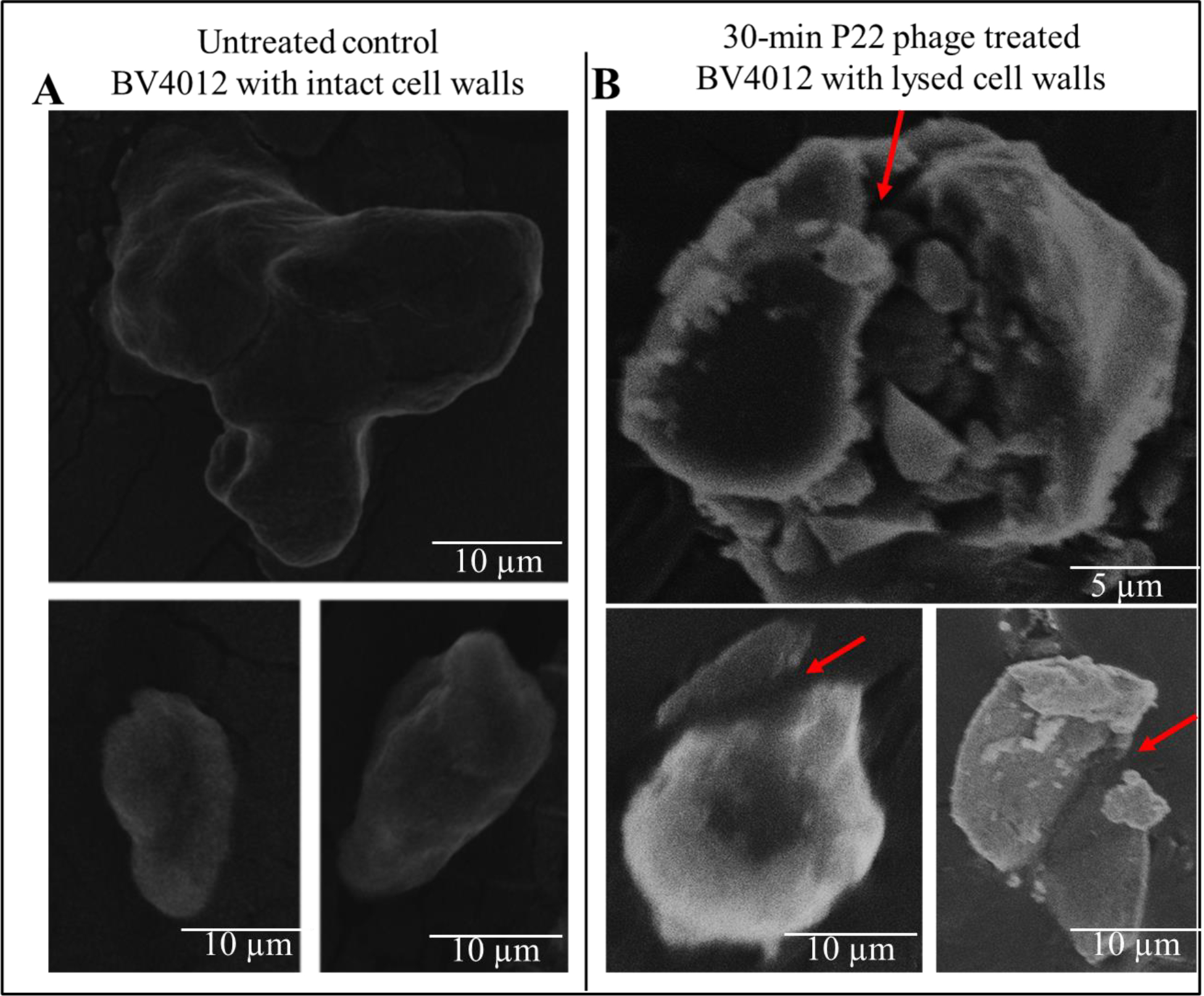
SEM images of *S. typhimurium* (BV4012) depicting the comparison between untreated and P22 phage treated samples. **(A)** Untreated BV4012 *S. typhimurium* visualized through Scanning Electron Microscopy (SEM), depicting unbroken cell wall of *S. typhimurium*. **(D)** P22 treated BV4012 *S. typhimurium* visualized through Scanning Electron Microscopy (SEM) shows lysed cell walls. Red arrows shows cell wall lysis.

To verify the lytic effect of P22 phage on *S. typhimurium*, BV4012 samples were infected with P22 phage and allowed to incubate at 37 °C. Samples were then subjected to SEM analysis. As shown in the untreated control samples (Fig. 2A), the morphologies of the bacteria shown unbroken cell wall, hence the absence of lysis. The P22 phage treated samples however showed bacteria with broken cell walls (Fig. 2B) indicating the lysis of BV4012.

### 3.3 P22 Phage Efficacy Under Varied pH

**Figure 3:**
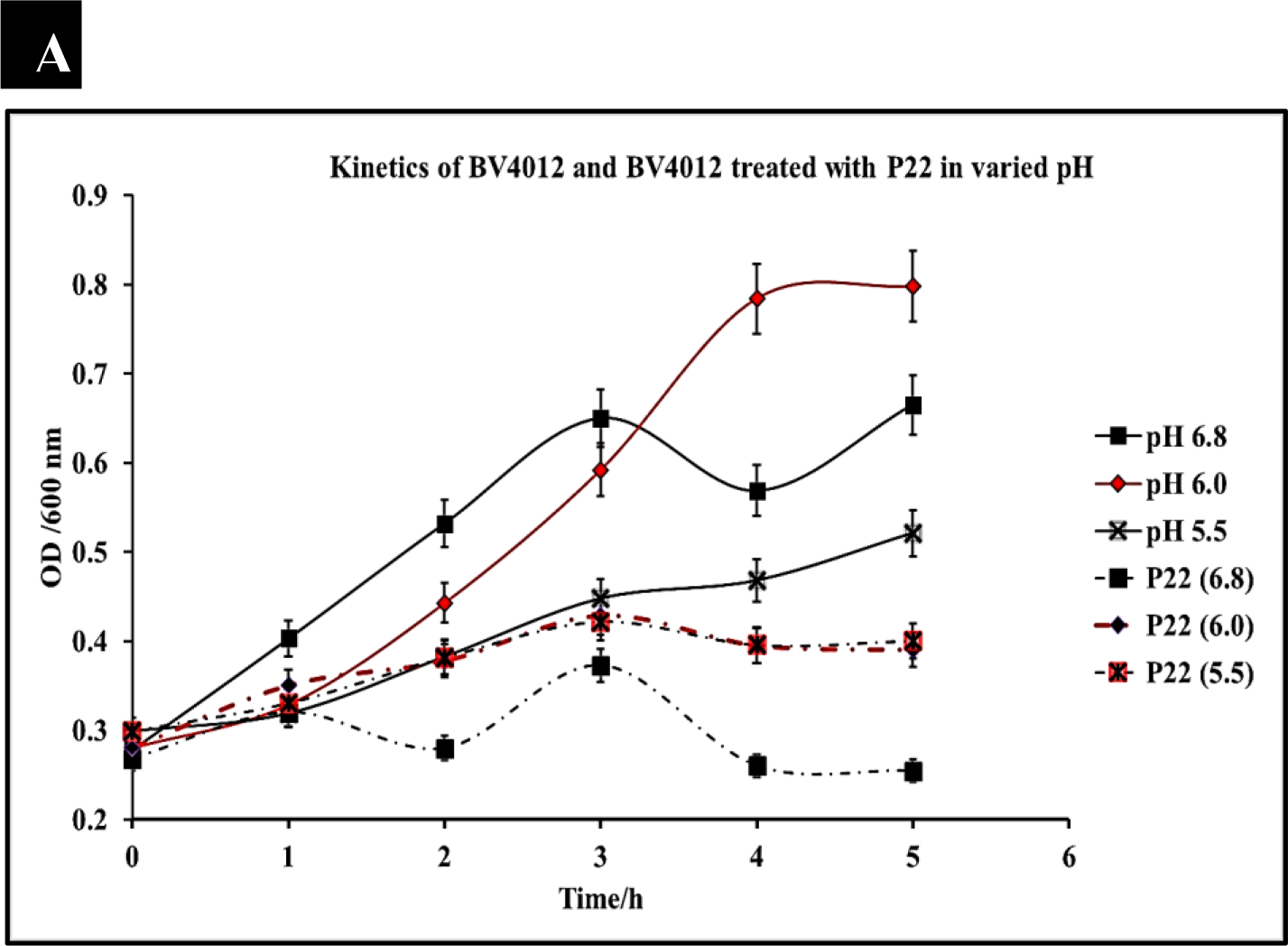

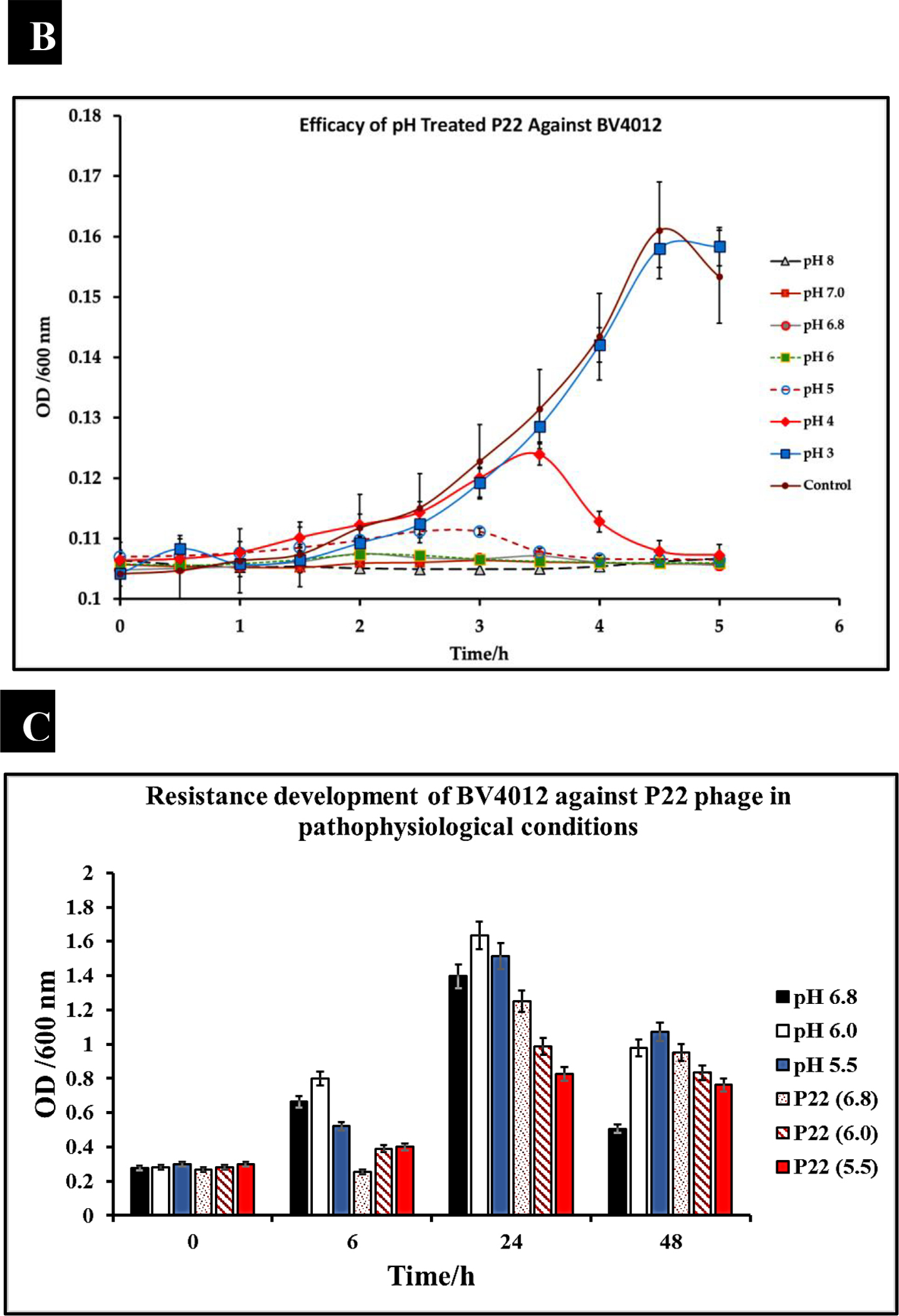
P22 phage efficacy against *S. typhimurium* under varied pH. *S. typhimurium* was cultured in varied pH conditions of 5.5, 6.0, and 6.8 and then treated with P22 phage. Three replicate OD600 were recorded hourly for 5 hours. **(A)** Efficacy of P22 against BV4012 in varied pH. BV4012 was cultured in varied pH condition of 5.5, 6.0, and 6.8 and then treated with P22 phage. Three replicate samples OD600 were recorded hourly for 5 hours **(B).** Efficacy of pH treated P22 against BV4012. P22 phage was exposed to pH varying from 3–8 and then introduced into BV4012 cultures. The OD600 was recorded hourly for 5 hours. **(C).** Resistance Development of BV4012 against P22 in pathophysiological conditions. BV4012 was cultured in pH of 5.5, 6.0, and 6.8 and then treated with P22 phage. Three replicate OD600 were recorded over a 48-hour period.

*S. typhimurium* infections are typically localized in the small and large intestine of the human body. This environment is characterized by acidic pH [33]. To consider P22 phage as a potential therapeutic, the phage must be able to function within these environmental constraints. We evaluated the efficiency of our phage P22 under pathophysiological conditions (pH ranging from 5–7) consistent with that present in human intestines. We cultured *S. typhimurium* at the pH of 5.5, 6.0, and 7 and measured optical density over 5 h to confirm bacterial growth at the pH of 5.5, 6.0, and 6.8. *S. typhimurium* (BV4012) cultures were then seeded into media at pH 5.5, 6.0, or 6.8 and then treated with P22 phage at 10 MOI. Optical densities recorded over 5 h determined that there was no functional loss of the phage in the acidic conditions and remained effective in reducing the growth of *S. typhimurium* (Fig. 3A).

To further examine the effect of pH on P22 phage functionality we exposed P22 phage alone to pH conditions ranging from 3 to 8 and then examined their efficacy against BV4012 in standard conditions. This study would determine that P22 could retain functionality and lyse BV4012 after exposure to pH conditions ranging from 8 down to 4. This data suggests that P22 is stable at these conditions as well as not susceptible to pH associated with pathophysiological conditions (Fig. 3B).

An important aspect of bacterial infection is the persistence of infections and the potential development of resistance associated with this persistence. We observed this development of resistance for 48 h to determine if there was any association between pH and rate of resistance development (Fig. 3C). Our results suggest that 24 h after treatment, there is a slight resurgence of growth, suggesting resistance development. However, preceding results 48 h after treatment still suggest that P22 is effective in reducing *S. typhimurium* growth.

### 3.4 P22 Phage Effective Against S. typhimurium in Stationary Phase

**Figure 4:**
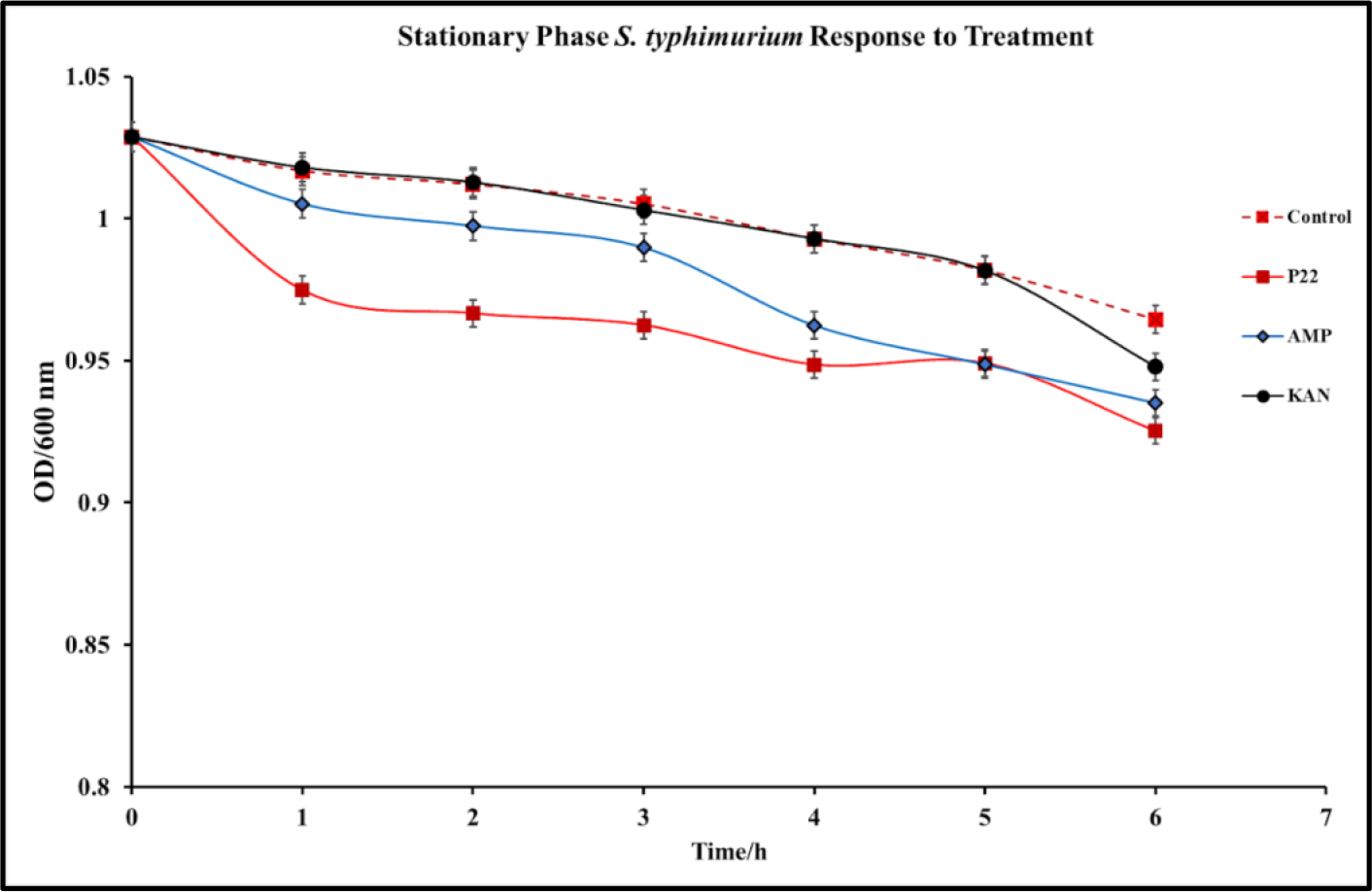
P22 phage efficacy against Stationary Phase *S. typhimurium* (BV4012). BV4012 was grown to an OD>1 and then treated with P22 phage, kanamycin, or ampicillin. Three replicate samples OD600 were recorded hourly over 6 hours.

In cases of infection, following the log phase of *S. typhimurium*, a stationary phase represented by slowed growth rate and often a non-replicative state occurs. This state is typically conducive to the formation of biofilms within the intestinal system [34]. This state presents a major issue for antibiotic treatment methods that are only effective against bacteria in the replicative state [35]. Understanding P22 phage activity against *S. typhimurium* in the stationary phase is an important aspect of determining the viability of the phage against *S. typhimurium* infections. To determine the efficacy of P22 phage against *S. typhimurium* in the stationary phase, *S. typhimurium* cultures were grown to an OD>10. The stationary *S. typhimurium* cultures were then infected with P22 phage, and optical density was observed over 24 h. Significant reduction in OD was observed in phage treated samples 24 h after treatment (Fig. 4). This study confirms the antibacterial capabilities of P22 phage against *S. typhimurium* at the stationary phase. Following 48 h of incubation, no regrowth was detected, suggesting that *S. typhimurium* did not develop resistance.

### 3.5 Efficacy of Ampicillin and Kanamycin Against S. typhimurium

**Figure 5:**
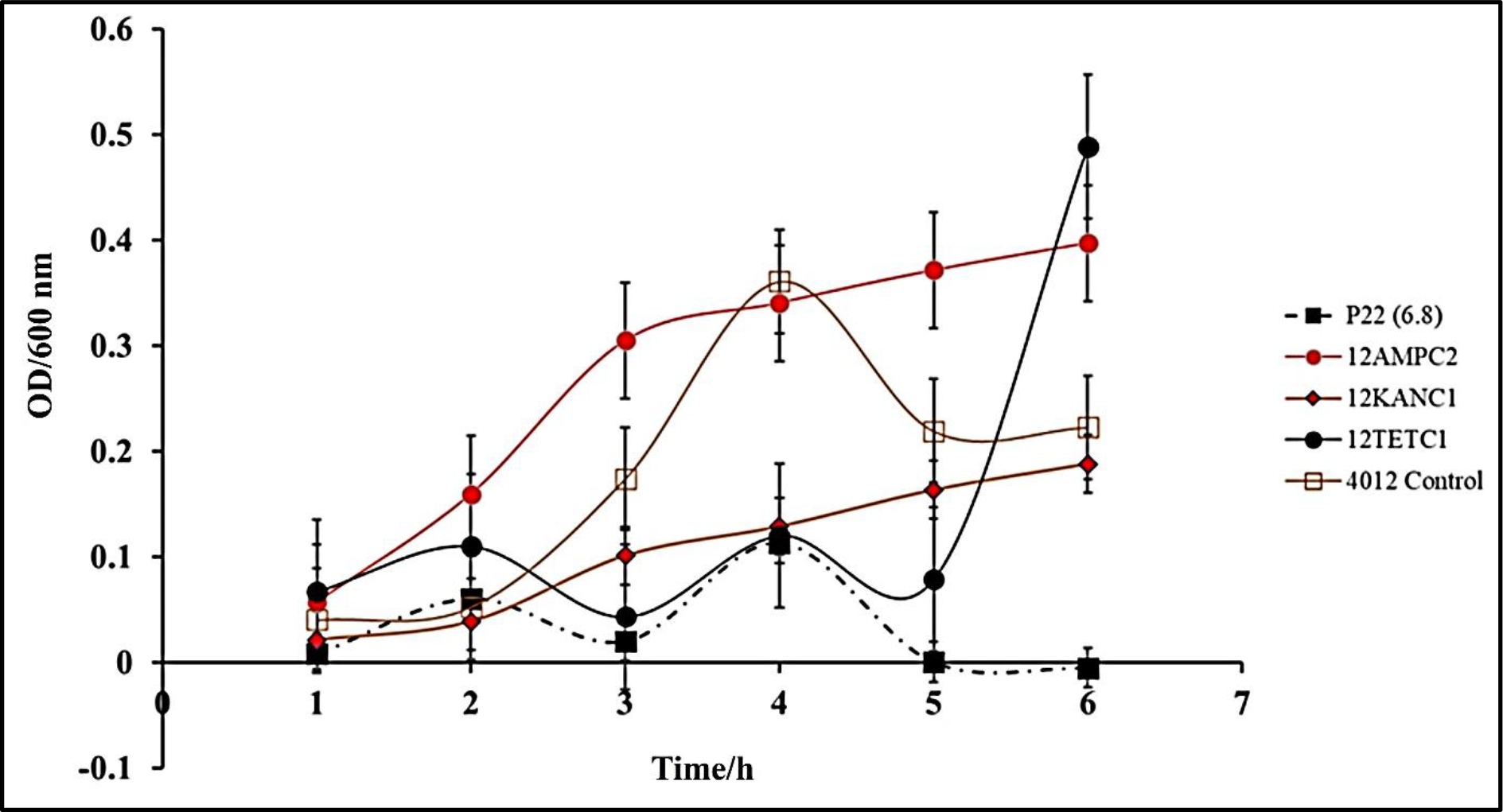
Comparative Efficacy of P22 phage and Antibiotic Treatments of *S. typhimurium* (BV4012) at normal physiological pH. BV4012 was cultured and treated with either P22, ampicillin, kanamycin, or tetracycline. Three replicate samples OD600 were recorded hourly for 6 hours to observe bacterial growth.

*Salmonella* infections are conventionally treated with a combination of common antibiotics including ampicillin or kanamycin but developed resistance to these treatments has become more prevalent [36]. To compare conventional antibiotic treatment to our novel phage treatment, comparative kinetic studies were conducted. Firstly, we determined minimal inhibitory concentrations for Ampicillin (0.5 μg/mL) and Kanamycin (0.5 μg/mL). Cultures were treated with the MIC of the respective antibiotic at the lag phase. Kinetics following treatment with either P22 phage at MOI of 1, ampicillin, or kanamycin revealed that the antibacterial function of P22 was significantly higher than that of either antibiotic account for a drastic decrease in the OD600 of *S. typhimurium* (Fig. 5).

### 3.6 Synergistic Characteristics of Phage-Antibiotic Co-Therapy

To observe the potential synergistic characteristics of phage-antibiotic co-therapy against *S. typhimurium*, checkerboard assays were conducted. *S. typhimurium* cultures (BV4012) were treated with antibiotics at concentrations of 50 μg/mL, 5 μg/mL, 0.5 μg/mL, or 0.05 μg/mL and P22 phage at MOIs of 10, 1, 0.1, or 0.01 during the lag phase. The optical density of the samples was recorded over 24 h. In comparison to ampicillin treated *S. typhimurium* cultures, cultures treated with ampicillin and P22 at MOIs of 0.1 and 1 resulted in greater inhibition of growth than ampicillin alone over the span of 24 h (Fig. 6A.). In comparison to kanamycin treated *S. typhimurium* cultures, cultures treated with kanamycin and 0.1 or 1 MOI of P22 resulted in greater inhibition of growth than kanamycin alone over the span of 24 h (Fig. 6B.). These results suggest a synergistic interaction between the ampicillin with the P22 phage at these concentrations. This is novel in the P22 phage system and similar work has been demonstrated in numerous other studies of bacterial infections such as *P. aeruginosa* and *S. aureus* [37, 38]. The correlation between increased growth reduction and MOIs of 0.1 and 1 of P22 suggest phage-antibiotic synergy. These antibiotics might affect cell lysis through targeting the cell membrane of *Salmonella* cells. This mechanism paired with the lytic nature of P22 provides synergistic killing activity against *S. typhimurium*. A major benefit of co-therapy that is demonstrated through these results is the reduced development of resistance to both antibacterial agents present when the bacteria are treated within the lag phase.

### 3.7 P22 Phage exhibits bacteriostatic behavior against Antibiotic-Resistant S. typhimurium

**Figure 7:**
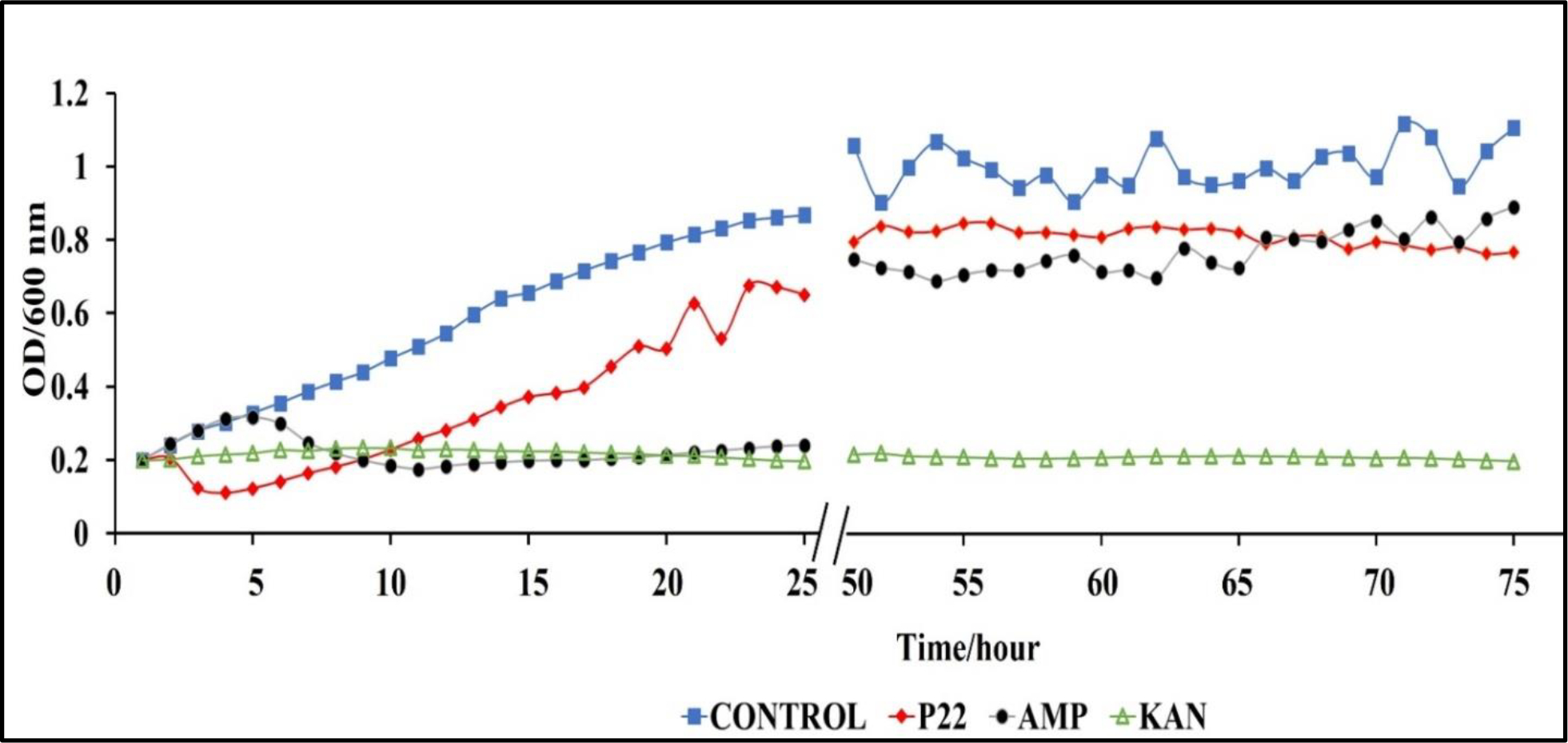
Rechallenge of *S. typhimurium* (BV4012) with P22 phage over 74 h. BV4012 was first treated with P22 phage, ampicillin, or kanamycin. Upon development of resistance, signified by increase OD600 after 25 hours, all samples were rechallenged with P22 phage and the OD600 was recorded hourly for 24 hours following treatment.

A major benefit of phages, over antibiotics, is their ability to complete lysis of antibiotic resistant bacterial strains [39–41]. In terms of clinical application, this significant role in the fight against antibiotic resistant bacterial infections. We conducted rechallenge experiments to observe the ability of our phage P22 to lyse *S. typhimurium* cultures with developed resistance to ampicillin or kanamycin. We inoculated BV4012 and BV4013 lag-phase cultures with either P22 phage, ampicillin or kanamycin and monitored the OD of the culture over 48 h until the OD began to steadily rise illustrating a developed resistance to the treatment. Once resistance was developed all cultures were inoculated with P22 phage at an MOI of 1 and the OD was measured over the preceding 24 h. Following 74 total h of incubation, we determined that the rechallenge of the cultures with P22 phage resulted in a significant reduction in the rate of *S. typhimurium* growth (Fig. 7). These results allowed us to infer that P22 phage can lyse the *S. typhimurium* cultures which developed resistance to ampicillin or kanamycin treatments.

## 4. Discussion

The development of novel treatment methods against pathogenic bacteria is essential to the preservation of public health. Bacteriophages have been shown to be promising as potential antibacterial agents against numerous pathogenic bacteria, including *Salmonella* spp., *Pseudomonas* spp., and *Staphylococcus* spp. [37, 38, 42]. While many phages have shown efficacy as potential therapeutics, extensive study of the effects of pathophysiological conditions on the efficacy of phages is limited. This study serves to determine the ability of P22 phage to maintain its functionality under several conditions associated with *S. typhimurium* infections in the small and large intestines. Understanding the limitations and boundaries of phages, such as P22 phage is essential to their application as therapeutic and preventative treatments.

For phage therapy to be successful, phages must retain their lytic function against their host in pathophysiological conditions and infect non-replicative stationary phase bacteria. This study focuses on *S. typhimurium*, a prevalent foodborne pathogen with an increased rate of antibiotic resistance over recent history [1–3]. There is an inherent need for the development of new therapy against *S. typhimurium* infections, since the efficacy of antibiotics is threatened and the potential for outbreak of antibiotic-resistant strains increases [1, 2, 10]. The phage P22, whose natural host is *S. typhimurium*, has been widely studied and utilized in some applications as a prophylactic [4, 42–46]. P22 exhibits a lytic lifestyle that is ideal for use as a potential therapeutic against *S. typhimurium* as well as been characterized as a having notable stability against several environmental conditions [4, 45]. The phage P22 is reliant on its incredibly stable tailspike protein (TSP) for the initiation of infection and lysis of *S. typhimurium* [44].

In this study, we first wanted to examine the kinetics associated with P22 phage infection of *S. typhimurium* at various growth stages, including lag-phase and log-phase. Our results illustrate the timeline of P22 phage infection and the associated reduction of bacterial growth and persistence in standard conditions. Treatment of *S. typhimurium* cultures at all three stages of growth resulted in a significant decrease in bacterial growth (Fig. 1A, 1B, 6A, 6B). These results serve to first prove the lytic ability of P22 phage against our target bacteria and secondly set a baseline for comparison of P22 phage performance in pathophysiological conditions. The lysis of *S. typhimurium* was demonstrated via scanning electron microscopy (Fig. 2B).

An important aspect in the study of any new novel agent is its comparison to standard therapeutic methods that are currently in place. In this study, we examined the comparative kinetics of P22 phage and two standard antibiotics: ampicillin and kanamycin, against *S. typhimurium*. An important distinction between bacteriophages and antibiotics is the versatility and abundance of phages in nature [47, 48]. Antibiotics, being man-made compounds require tedious design and development while also having low specificity and limited classes. There are estimated to be approximately 10^31^ bacterial and archaeal viruses present in the biosphere with over 500 of these phages’ genomes having been fully sequenced [49].

The ability to withstand acidic pH conditions is a major requirement of any antibacterial agent. The proposed use of P22 as a therapeutic against *S. typhimurium* requires it to withstand the pH conditions of the gastrointestinal tract where this bacterium typically colonizes [27, 28]. P22 phage was able to retain lytic function and reduce bacterial growth at pHs of 5.5, 6.0, and 6.8 (Fig. 3A). These pHs are consistent with the physiological conditions associated with the small and large intestines of humans. More importantly, these results confirm the ability of P22 phage to withstand physiological conditions found within these organs and retain its antibacterial function. Our study went further to also determine the pH limitations of P22 phage. It was determined that P22 phage even after extended exposure to media at pH 4–8 was able to retain antibacterial function against *S. typhimurium* (Fig. 3B). The pH threshold of P22 phage was determined in this study as well and suggested a loss of functionality at a pH of 3 (Fig. 3B). Resistance development was also examined in this study over a 48-h period revealing that after 48 h of P22 phage treatment, there was a slight resurgence of growth across all pH levels (Fig. 3C). Resistance development was significantly higher at the pH of 5.5 and 6.0 in both treated and untreated samples suggesting a correlation between resistance development and acidic pH. The development of resistance by bacteria when exposed to acidic pH has been recreated in studies of several other bacteria [50, 51]. Overall, our study concludes that P22 phage is effective in drastically reducing the growth of *S. typhimurium* within the first 24 h of administration even in acidic conditions which are similar to the physiological conditions of the gastrointestinal tract.

Another pathophysiological condition that was also examined in this study is the efficacy of P22 against *S. typhimurium* in the stationary phase. Infections of the intestinal system by *S. typhimurium* in the non-replicative stage is a major concern in the clinical setting [52]. Stationary phase bacterial infections typically are associated with the development of biofilms and antibiotic resistance [34]. Considering this, it is essential to develop methods for these difficult to treat infections and our results suggest that P22 phage shows potential as a co-therapy or alternative treatment (Fig 4). Kinetic study of P22 phage treated stationary-phase *S. typhimurium* shows that P22 phage inhibited bacterial growth at a rate higher than that of ampicillin or kanamycin over a 6-h period. Extended kinetics over a 48-h period confirmed that P22 treatment also did not result in regrowth of *S. typhimurium*.

Results show that P22 phage drastically stunted bacterial growth and nearly eliminated *S. typhimuri*um completely after 6 h (Fig 5). In comparison to ampicillin and kanamycin, P22 phage performed significantly better in reduction of *S. typhimurium*. In this study we also examined the potential synergy between P22 phage and the antibiotics ampicillin and kanamycin. The synergistic characteristics of phage-antibiotic co-therapy has been suggested in numerous studies and is a promising incorporation of phage therapy to combat resistant infections [22–24, 53]. Our results suggest that P22 at MOIs of 1 and 0.1 paired with ampicillin or kanamycin reduces growth of *S. typhimurium* at a rate greater than either antibiotic individually (Fig. 6).

While phages such as P22 show promise as potential therapeutics, it is important to consider the ability of bacterial species to develop resistance against phage treatments [54, 55]. Our studies did determine that there were cases of partial resistance against P22 by *S. typhimurium*. This resistance was observed in the form of increased bacterial growth after extended exposure of the bacteria to P22. The mechanism of resistance was not clear and requires further study. Phage resistance is mediated by bacteria in several ways including but not limited to receptor mutation, blockage of phage DNA injection, hypermutable loci, cleavage of phage DNA, abortive infection, R-M system, DISARM system, or CRISPR-Cas system [56–59].

A limitation of our study is the use of fast-growing laboratory use *S. typhimurium* (BV4012). While this strain is ideal for our study, it may not translate to naturally occurring *S. typhimurium* infections with variations in growth rate. It was demonstrated that P22 is effective against *S. typhimurium*, however after extended exposure, the development of resistance raises concerns about the use of P22 phage and the threat of infection resurgence. Further study of the resistance mechanisms possessed by *S. typhimurium* against P22 phage is essential to gaining a full understanding of this interaction. While we showed that P22 is effective *in vitro* against *S. typhimurium* cells, we did not explore phage delivery methods *in vivo*. The delivery of P22 phage to the location of infection is essential to the success of therapy, and an area that requires further study. Another major challenge of phage therapy is the human immunological response. Studies show that there is an immunological response to phage treatments and that this response can be in ways synergistic in the elimination of acute and chronic bacterial infections [60–63].

## 5. Conclusion

In summary, we demonstrated the efficacy of the phage P22 under pathophysiological conditions associated with *S. typhimurium*. These conditions included lag-phase, log-phase, and stationary phase of *S. typhimurium* as well under various pH ranges (4–8). Additionally, we showed that P22 phage is effective killing S. typhimurium, and the phage seemed to perform comparably to standard antibiotic’s such as ampicillin and kanamycin in killing *S. typhimurium*. We examined the synergistic potential of P22 phage at MOIs of 10, 1, 0.1 and 0.01 with ampicillin or kanamycin at concentrations of 50 μg/mL, 5 μg/mL, 0.5 μg/mL, 0.05 μg/mL against *S. typhimurium* cultures. The best synergistic effect was observed at phage MOI of 0.1 and kanamycin concentrations of 0.5 and 0.05 μg/mL. Whereas the best synergistic effect was observed at MOI of 1 of P22 phage and 0.5 μg/mL of ampicillin co-treatment.

## Figures and Tables

**Figure 6A:**
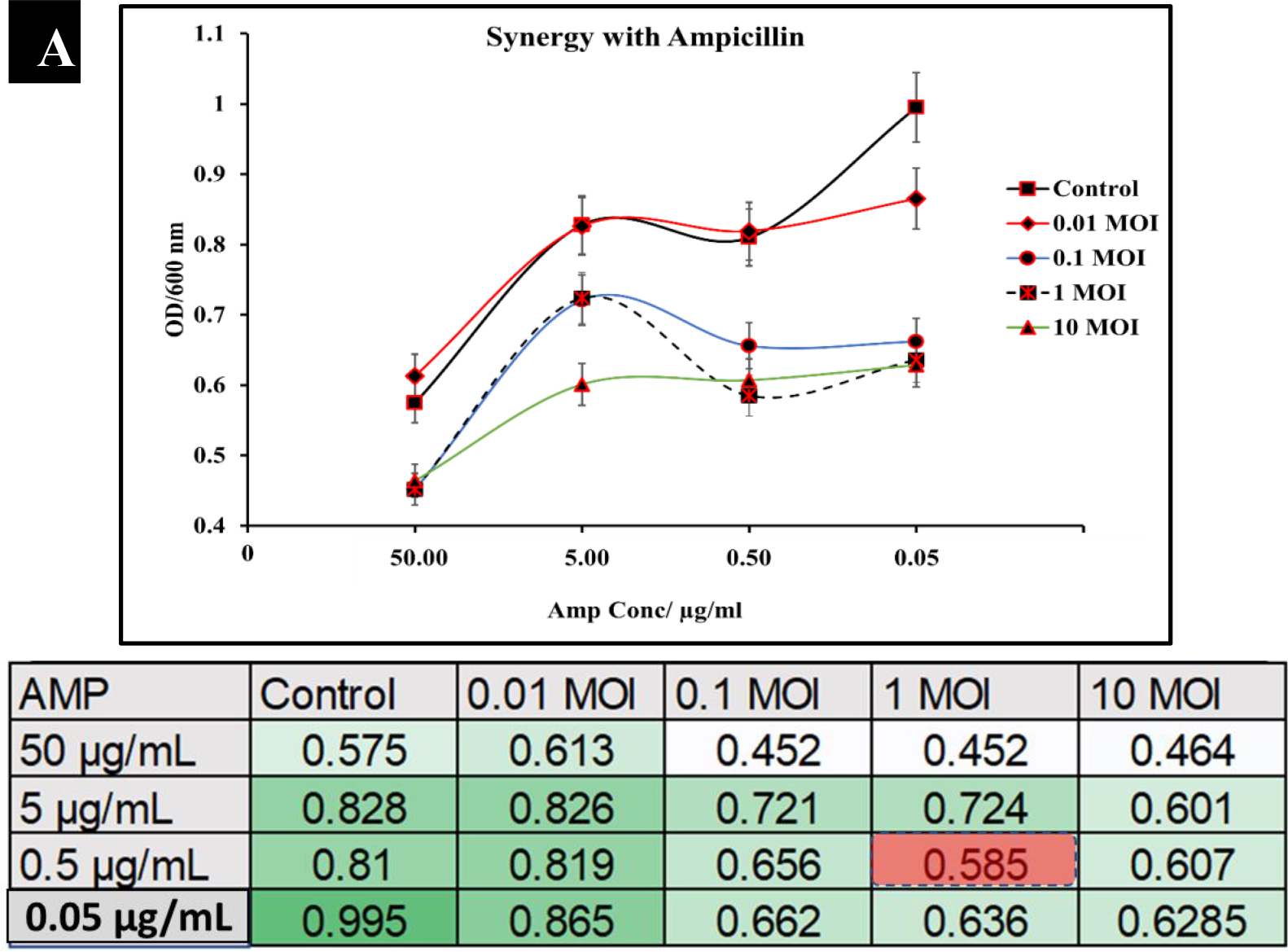
Synergistic analysis of ampicillin and P22 phage co-treatment of *S. typhimurium*. Data presented as mean ±Standard deviation in the chart, whereas the table shows the checkerboard representation of the chart. The best synergistic effect was observed at MOI of 1 of P22 phage and 0.5 μg/mL of ampicillin co-treatment.

**Figure 6B:**
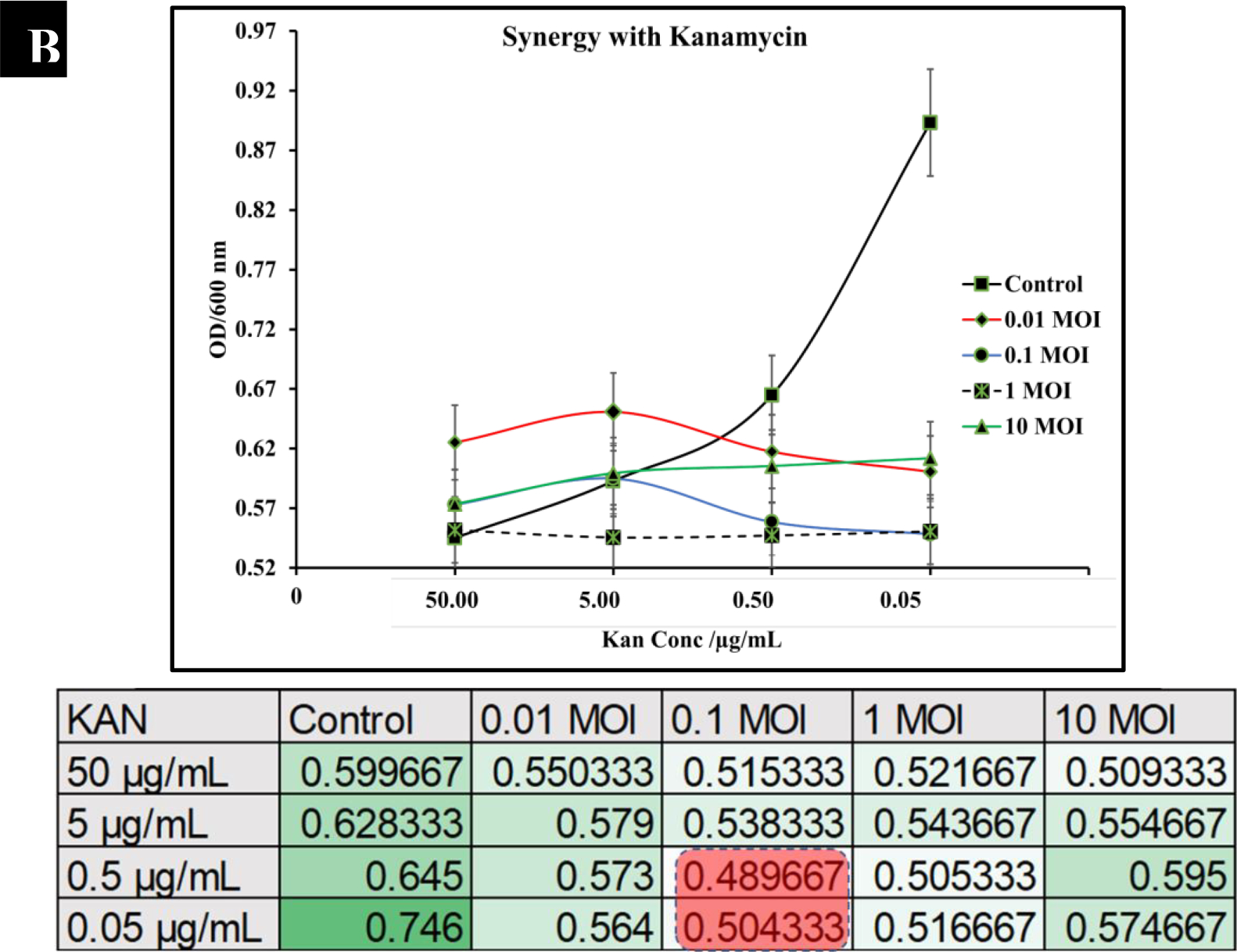
Synergistic analysis of kanamycin and P22 phage co-treatment of *S. typhimurium*. The best synergistic effect was observed at phage MOI of 0.1 and kanamycin concentrations of 0.5 and 0.05 μg/mL.
